# Author Correction: Structure and electromechanical coupling of a voltage-gated Na^+^/H^+^ exchanger

**DOI:** 10.1038/s41586-023-06880-1

**Published:** 2023-11-22

**Authors:** Hyunku Yeo, Ved Mehta, Ashutosh Gulati, David Drew

**Affiliations:** Department of Biochemistry and Biophysics, Science for Life Laboratory, Stockholm University, Stockholm, Sweden

**Keywords:** Cryoelectron microscopy, Reproductive biology, Permeation and transport

Correction to: *Nature* 10.1038/s41586-023-06518-2 Published online 25 October 2023

In the version of the article initially published, there was an error in the Data availability section where the Protein Data Bank and Electron Microscopy Data Bank accessions “8OTW and EMD-17185” previously read “80TW and EMD-17165”. This has now been corrected in the HTML and PDF versions of the article.

