# Supplementary material for: Structure and electromechanical coupling of a voltage-gated Na+/H+ exchanger
Source: Nature. 2023 Oct 25;623(7985):193–201. doi: 10.1038/s41586-023-06518-2 (PMC10620092; doi:10.1038/s41586-023-06518-2)
Supplement: Supplementary file 1 — Supplementary Information [file 41586_2023_6518_MOESM1_ESM.pdf]

nature portfolio

https://doi.org/10.1038/s41586-023-06518-2

Supplementary information

Structure and electromechanical coupling of a voltage-gated Na^+/H^+ exchanger

In the format provided by the authors and unedited

Structure and electromechanical coupling of the voltage-gated Na^+/H^+ exchanger SLC9C1

Hyunku Yeo[1*], Ved Mehta[1*], Ashutosh Gulati[1*], David Drew[1#]

[*]Equal contribution; [#]Corresponding author

[1]Department of Biochemistry and Biophysics, Science for Life Laboratory, Stockholm University, SE–106 91 Stockholm, Sweden.

Supplementary Information file containing:
Supplementary Figs. 1–6
Supplementary Video Legends 1-3

Table of contents

Supplementary Fig 1. – p2

Supplementary Fig 2. – p5

Supplementary Fig 3. – p6

Supplementary Fig 4. – p8

Supplementary Fig 5. – p10

Supplementary Fig 6. - p12

Supplementary Video Legends 1-3. – pg 14

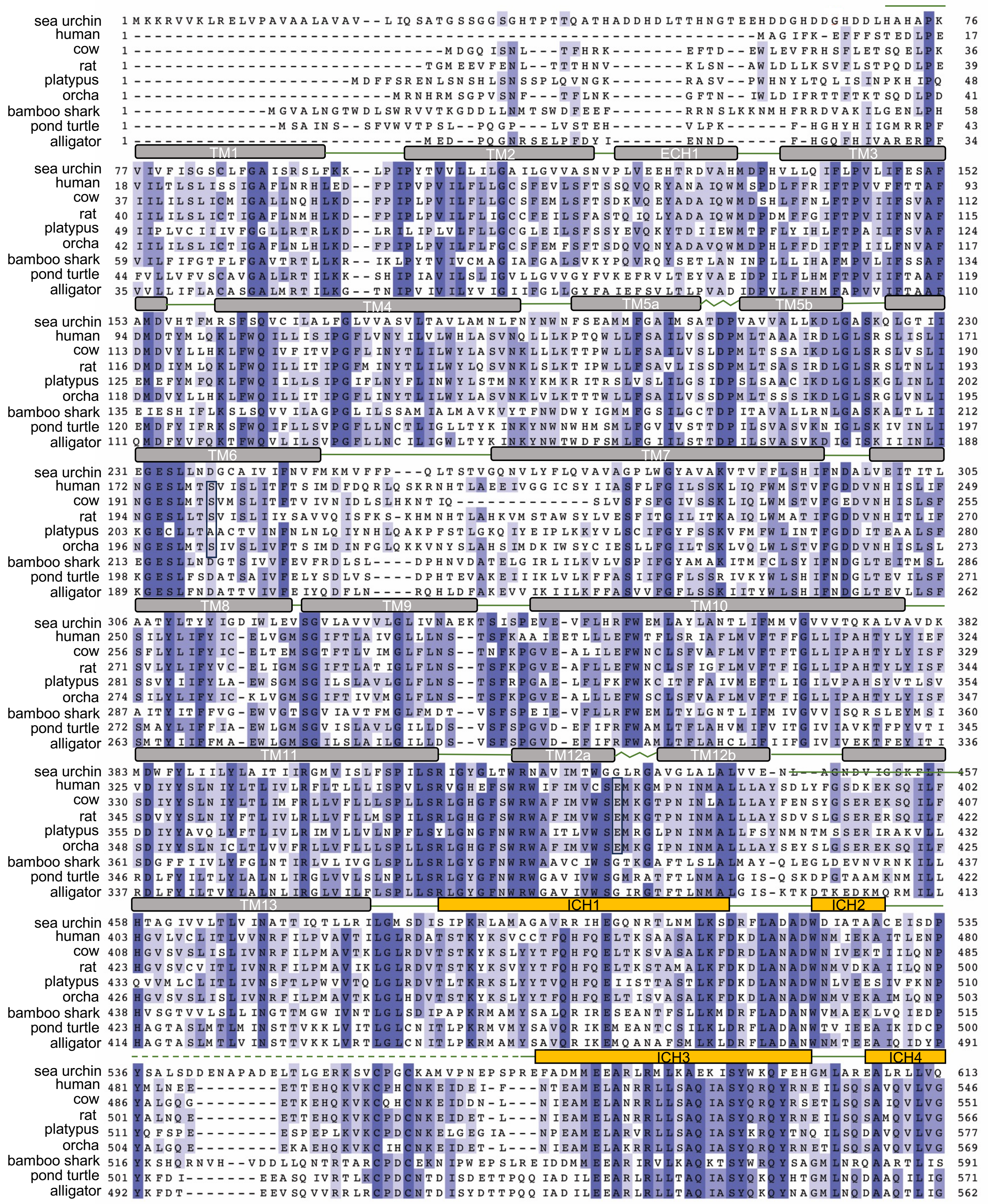
sea urchin
human
cow
rat
platypus
orcha
bamboo shark
pond turtle
alligator
TM1
TM2
ECH1
TM3
TM4
TM5a
TM5b
TM6
TM7
TM8
TM9
TM10
TM11
TM12a
TM12b
TM13
ICH1
ICH2
ICH3
ICH4

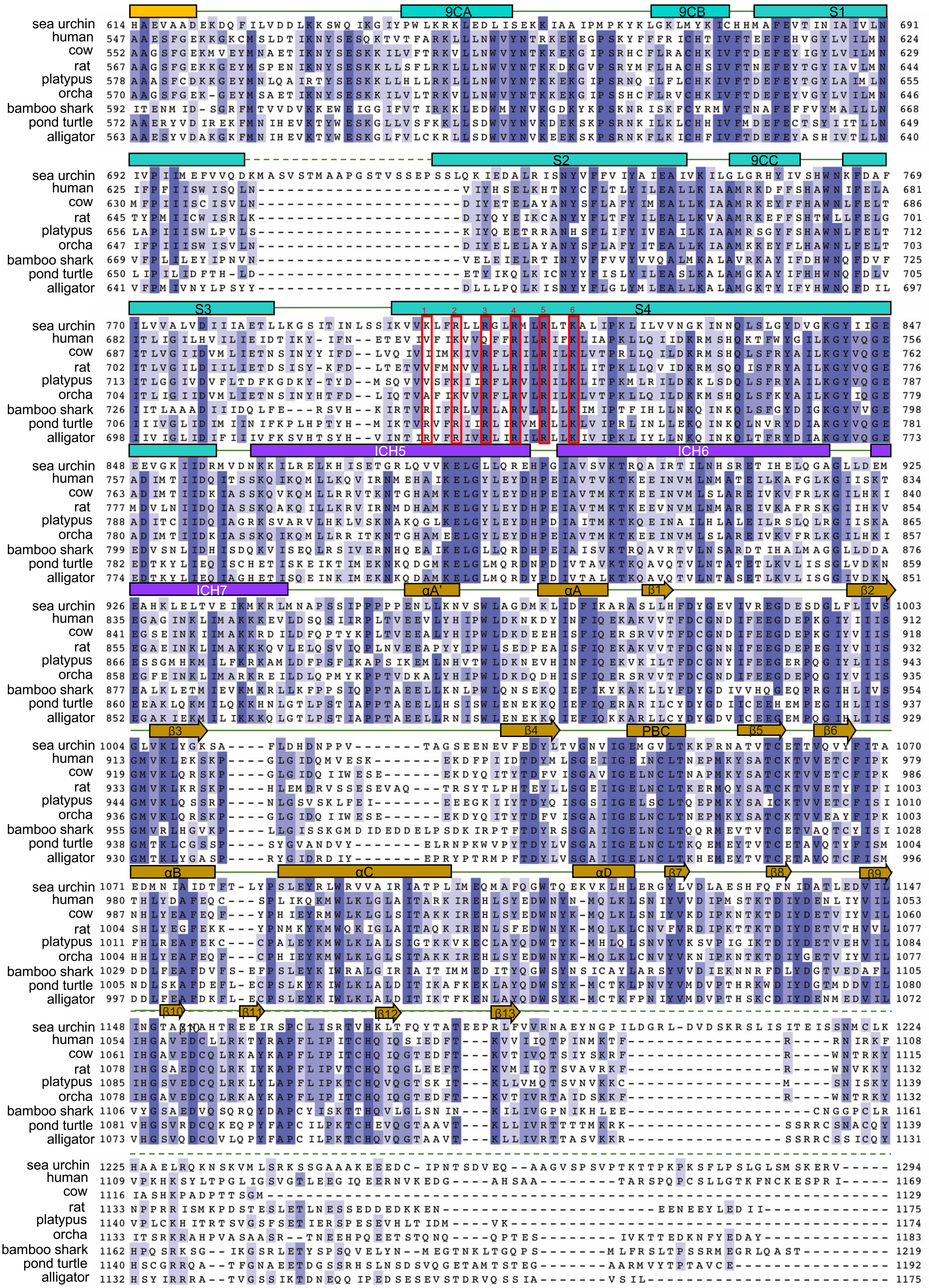
sea urchin
human
cow
rat
platypus
orcha
bamboo shark
pond turtle
alligator
9CA
9CB
S1
S2
9CC
S3
S4
1 2 3 4 5 6
ICH5
ICH6
ICH7
αA'
αA
β1
β2
β3
β4
PBC
β5
β6
αB
αC
αD
β7
β8
β9
β10
β11
β12
β13

Supplementary Fig. 1| Multiple sequence alignment of SLC9C1 homologues. Multiple sequence alignment of SLC9C1 homologues was performed using the Clustal[1] omega server and conservation scores displayed using Jalview with conservation scores ranging from light blue to dark blue corresponding to conservation scores ranging from low to high respectively. Secondary structural features of *sea urchin* SLC9C1 are depicted along the protein sequence as labelled rectangles as coloured in Fig. 3a: Transmembrane (TM), Intracellular Helix (ICH), Segments (S) in VSD, connecting interfacial helices in VSD (9CA, 9CB, 9CC) and CNBD. The CNBD secondary structure elements (αA', αA-αC, β1- β6) and PBC (phosphate-binding cassette) are labelled as convention for CNBDs and shown in Extended Data Fig. 8a. The CNBDs in SLC9C1 has an additional helix (αD) and β–roll domain labelled (β7- β13). Dotted lines represent regions that were not able to be modelled due to poor map quality. Residues highlighted in red correspond to the GC residues R1 to K6 in *sea urchin* SLC9C1. SLC9A (NHE1-9) and SLC9B (NHA1-2) Na^+/H^+ exchangers have a strictly conserved ion-binding aspartic acid corresponding to D238 in *sea urchin* SLC9C1[2,3]. The ion-binding aspartate D238 is not conserved in the mammalian SLC9C1 members shown (black-box), and these members contain an additional glutamate residue in the TM12a-b breakpoint (black-box), which is likely to fulfil the same role in ion-coordination. The SLC9C1 homologues were selected to encompass a spread of increasing protein sequence identity between *sea urchin* and *human* SLC9C1. The UniProt ID of the various SLC9C1 homologues are as follows: A3RL54 (*Strongylocentrotus purpuratus*), Q4G0N8 (*Homo sapiens*), F1MQF9 (*Bos tarus*), A0A0G2K938 (*Rattus norvegicus*), XP_028938660.1 (*Ornithorhynchus anatinus*), XP_049566288.1 (*Orcinus orca*), XP_043562352.1 (*Chiloscyllium plagiosum*), XP_044872492.1 (*Mauremys mutica*) and XP_006266403.1 (*Alligator mississippiensis*).

a.

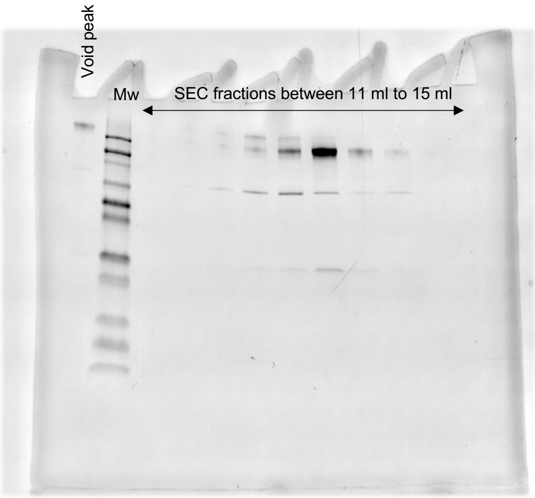

Cropped gel shown in
Extended Data Fig. 1a

b.

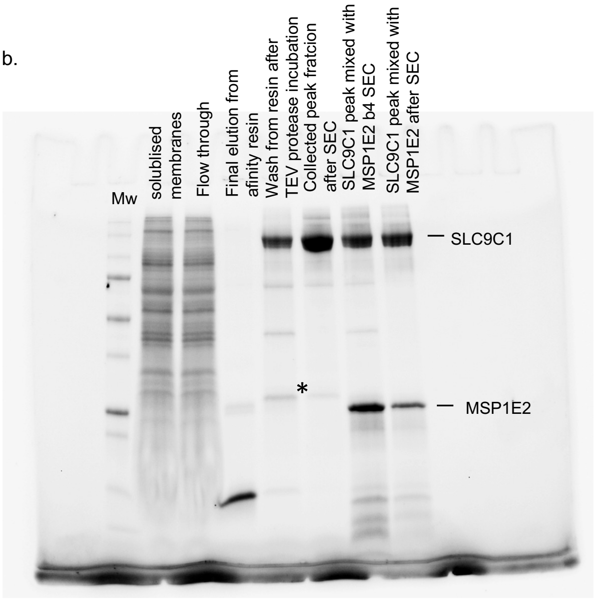

Cropped gel shown in
Extended Data Fig. 2a

*contaminant band

c.

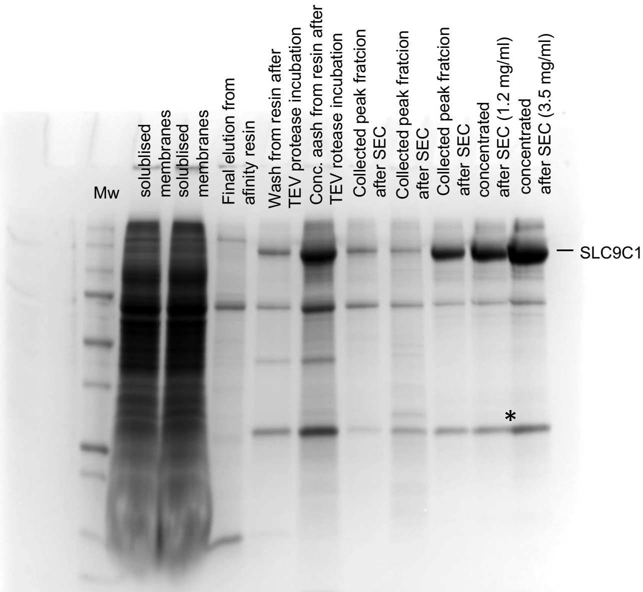

Cropped gel shpwn in
Extended data Fig. 9a

*contaminant band

d.

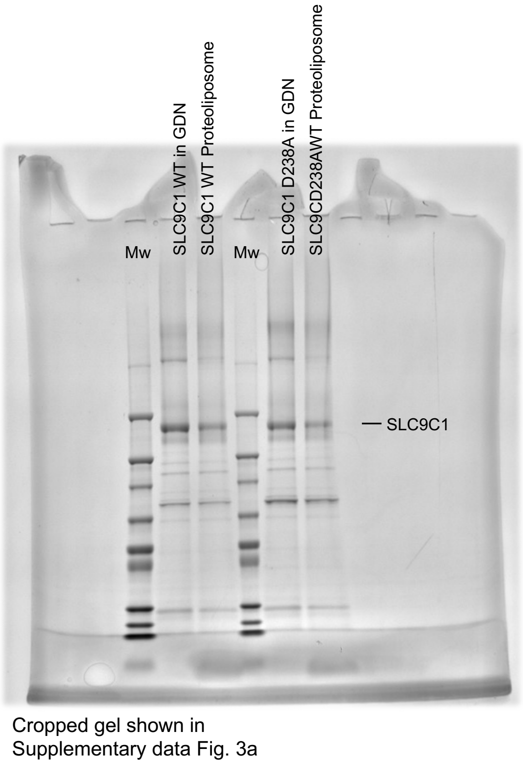

Cropped gel shown in
Supplementary data Fig. 3a

Supplementary Fig. 2| Uncropped Coomassie-stained SDS-gels.

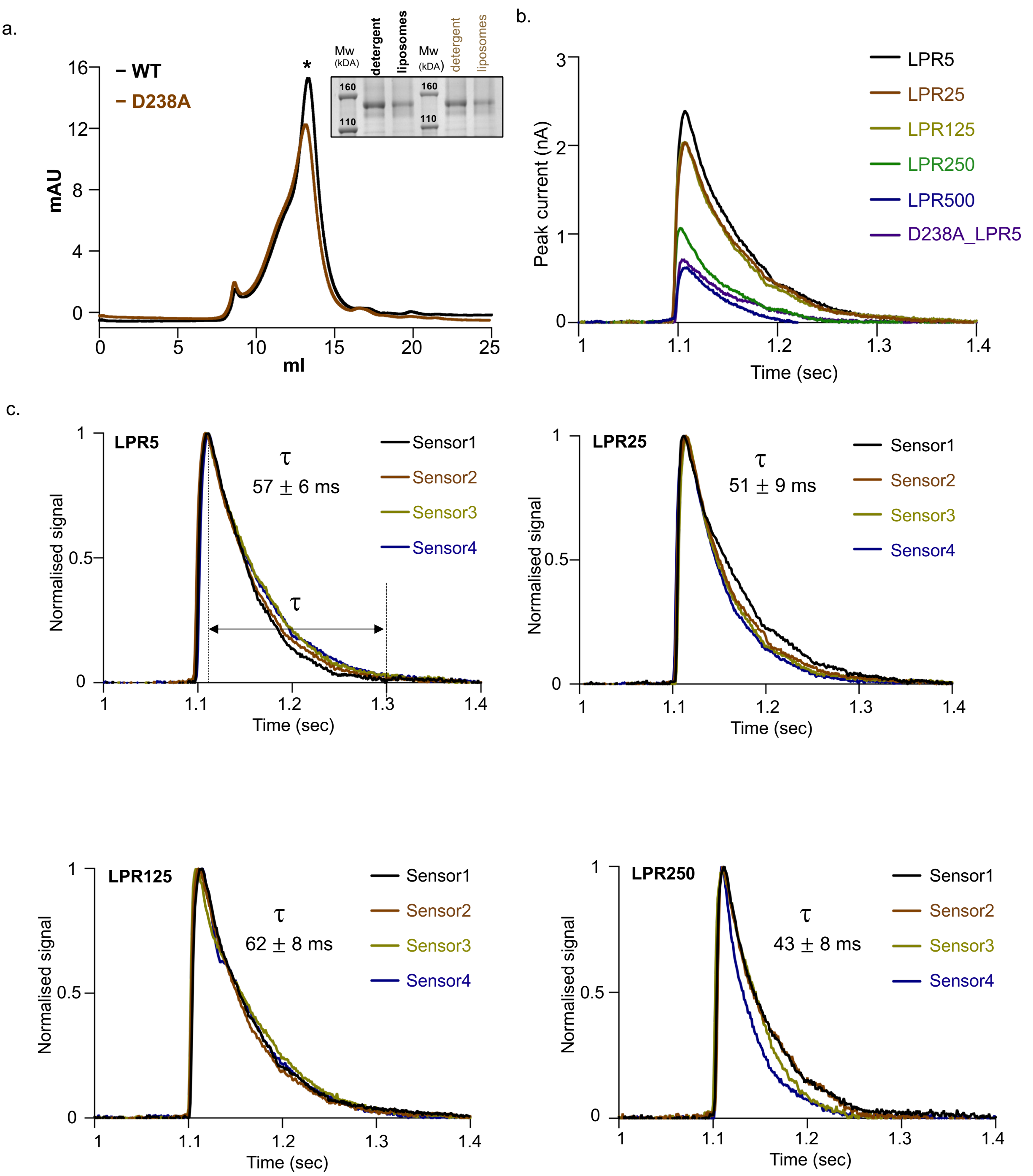

Supplementary Fig. 3| Solid State Membrane (SSM) based electrophysiology of sea *urchin* SLC9C1. **a.** Size-exclusion chromatography profiles of purified *sea urchin* SLC9C1 WT (black trace) and D238A variant (brown trace) used for reconstituted into liposomes made from yeast polar lipids (see Methods); triplicate independent purifications were performed with similar results. Inset: Coomassie-stained SDS-gel of purified SLC9C1 WT and D238A variant used for liposome reconstitution and the final amount in proteoliposomes at the highest LPR5. **b.** Representative transient currents of WT SLC9C1 containing liposomes after the addition of 80 mM NaCl, which were recorded under symmetrical pH 6.5 conditions with varied lipid-to-protein (LPR) ratios from LPR 500 to LPR 5. The response after the addition of 80 mM NaCl to the SLC9C1 D238A variant at LPR 5 is also shown. **c**. Replicates of normalised peak currents in response to 80 mM NaCl at symmetrical pH 6.5 for the varied lipid-to-protein (LPR)

ratios shown in Fig. 1c, inset. The average fast decay time (τ) is similar at different LPR values (values are the mean values ± s.d. of n = 4 independent sensors), a defining characteristic for transient currents generated by binding, rather than a transported charge[4]. The marginally lower average decay time value for LPR 250 is within the range of error for LPR 5 and could be explained by a larger variance in repeats, due to a lower current amplitude. For this reason, normalised peak currents for LPR 500 were not included as the signal was too weak for an accurate comparison i.e., LPR 500 gave similar current peak amplitudes as the D238A variant at LPR 5.

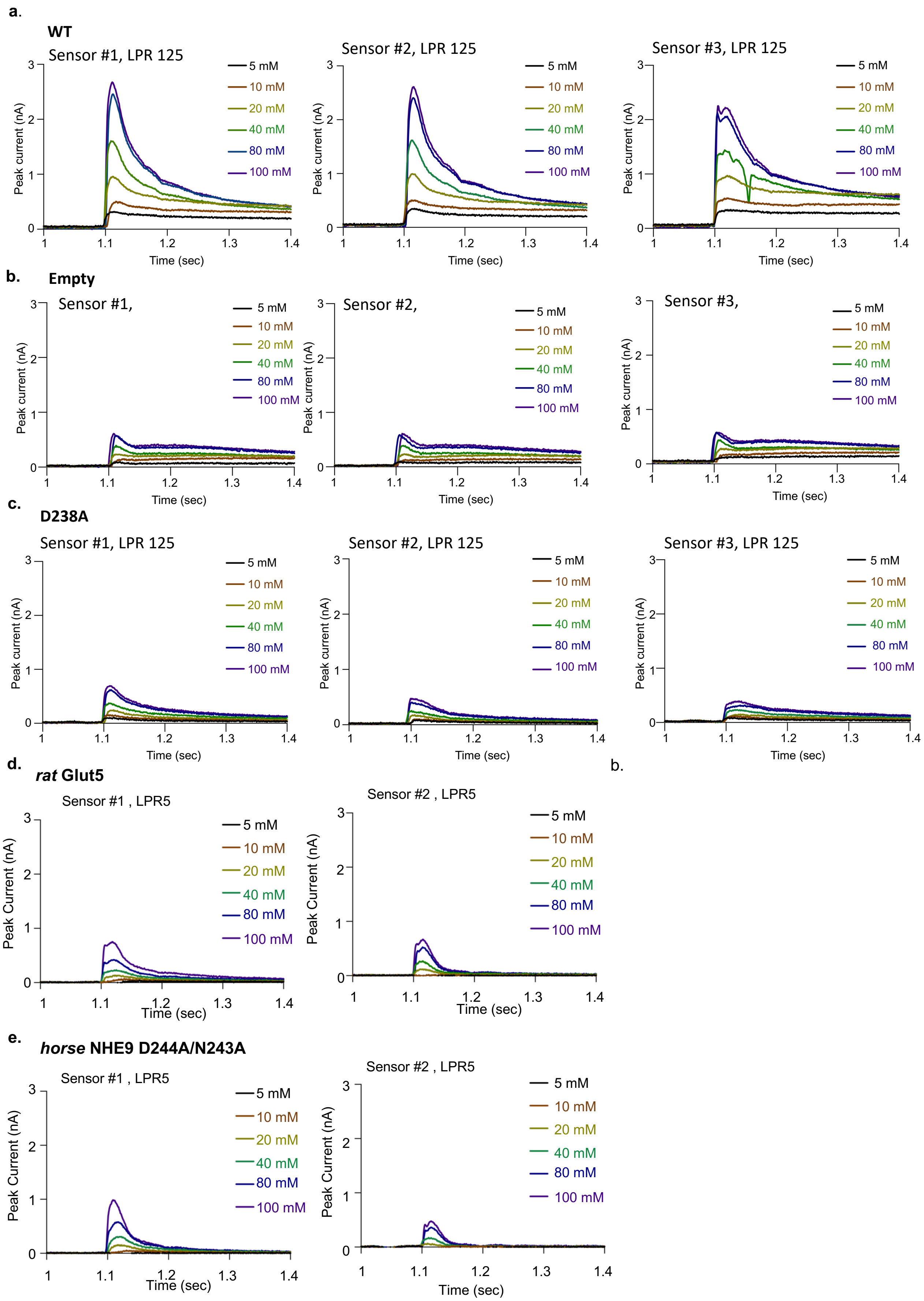
a.
WT
Sensor #1, LPR 125
Sensor #2, LPR 125
Sensor #3, LPR 125
b.
Empty
Sensor #1,
Sensor #2,
Sensor #3,
c.
D238A
Sensor #1, LPR 125
Sensor #2, LPR 125
Sensor #3, LPR 125
b.
d.
rat Glut5
Sensor #1 , LPR5
Sensor #2 , LPR5
e.
horse NHE9 D244A/N243A
Sensor #1 , LPR5
Sensor #2 , LPR5
5 mM
10 mM
20 mM
40 mM
80 mM
100 mM
Peak current (nA)
Peak Current (nA)
Time (sec)
0
1
2
3
1
1.1
1.2
1.3
1.4

Supplementary Fig. 4| Solid State Membrane (SSM) based electrophysiology for *sea urchin* SLC9C1. a. Representative transient current traces for each external Na^+ concentration are shown for SLC9C1 WT proteoliposomes with an LPR 125 with individual 1 mm sensors as labelled. **b**. As in a., for empty liposomes. **c**. As in a, for the SLC9C1 D238A variant. **d**. proteoliposomes with an LPR 5 for the fructose transporter GLUT5. **e**. As in a, for the horse NHE9 ion-binding aspartate variant D244A/N243A previously shown to abolish ion-exchange[5] with an LPR 5.

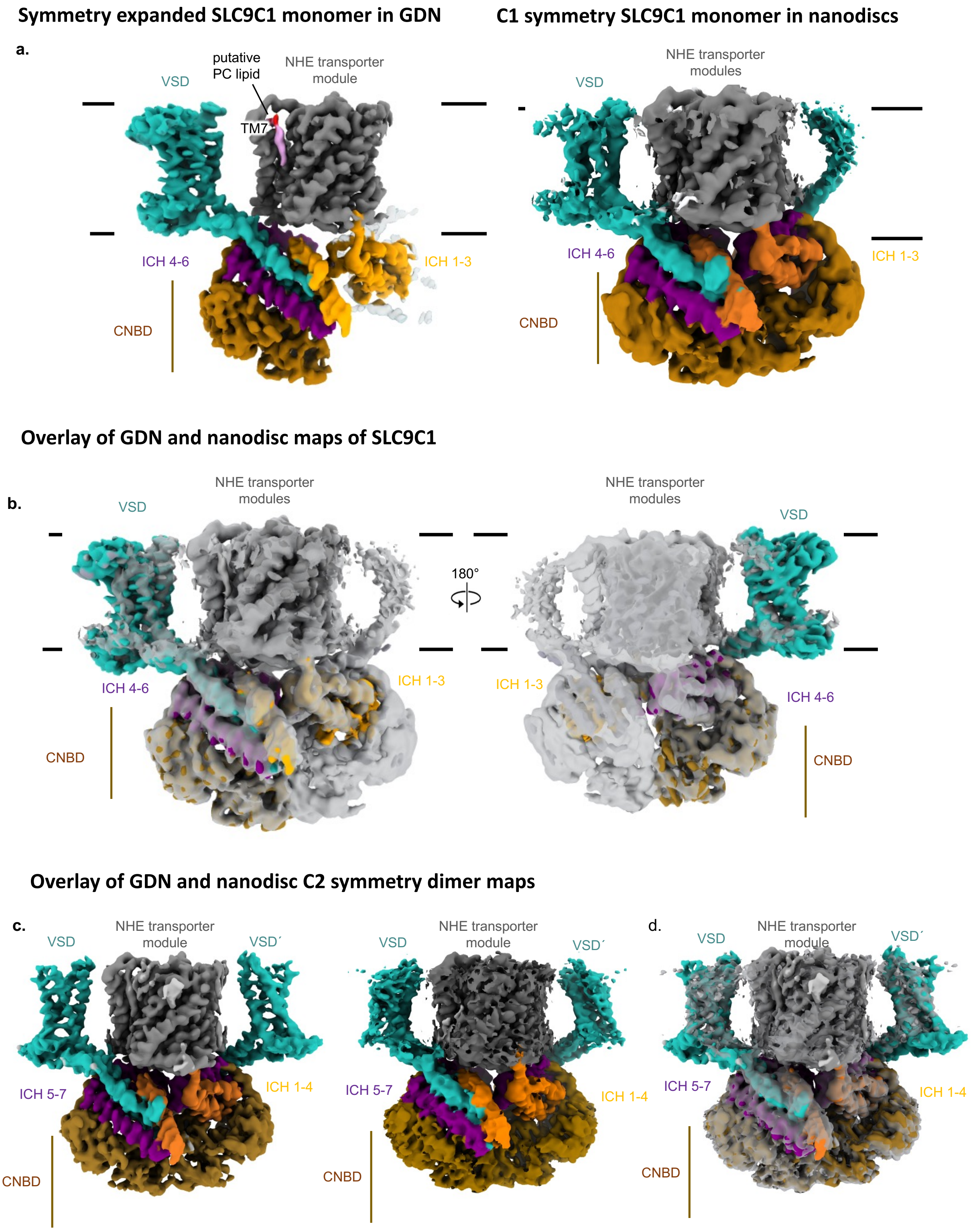

Supplementary Fig. 5| Comparing Cryo- EM maps of *sea urchin* SLC9C1 structures in detergent and nanodiscs, which show no clear differences in the positioning of VSDs relative to the SLC9C1 transporter module a. symmetry expanded monomer density of SLC9C1 in GDN (left) and C1 symmetry homodimer density in nanodiscs (right), which has been colored as in Fig. 1f and the PC lipid in the detergent structure is also highlighted (pink). **b.** Superimposition of the cryo-EM maps as shown in a., with the nanodisc density colored in gray. **c.** Cryo-EM density of SLC9C1 homodimer after C2 symmetry in detergent (left) and in

nanodisc (right). **d**. Overlay of the C2 symmetry cryo-EM density of the maps shown in c., with nanodisc maps in gray, highlighting the same positioning of both VSDs.

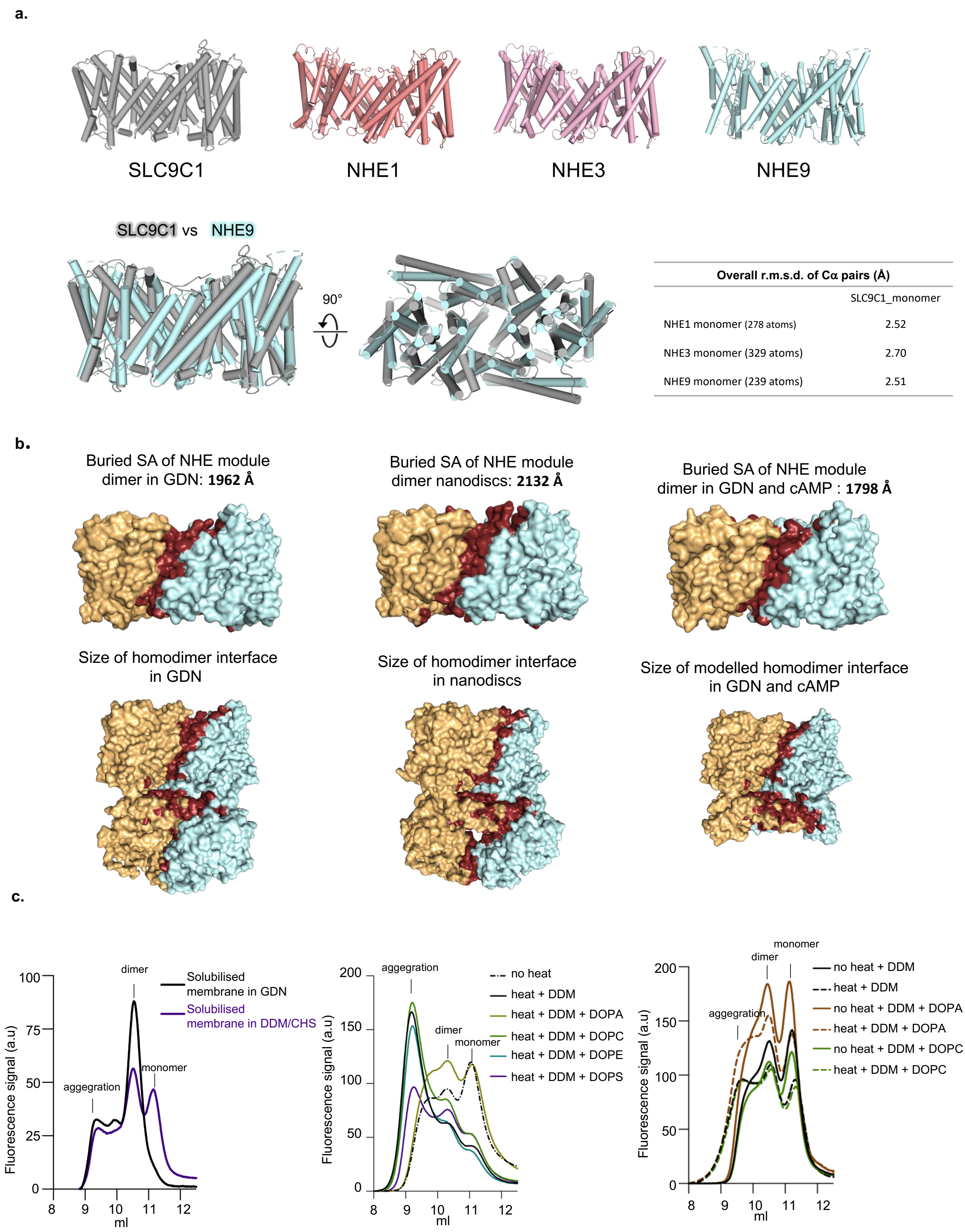

Supplementary Fig. 6| Structural comparison of sea urchin SLC9C1 with mammalian NHEs and lipid analysis. a. above: Cartoon representation of *sea urchin* SLC9C1 (gray), *human* NHE1(PDB ID: 7DSV, salmon), *human* NHE3 (PDB ID: 7X2U, pink) and *horse* NHE9 (PDB ID: 6Z3Y, cyan). below: Structural superimposition of the inward-facing *sea urchin* SLC9C1 homodimer structure (gray) with the inward-facing homodimer structure of *horse* NHE9 (cyan). The overall r.m.s. deviation (Å) of the Cα atoms of monomer structural

superimposition are further shown in the accompanying table. **b.** Buried surface area of homodimerization surfaces of the SLC9C1 structures in GDN (left), in nanodiscs (middle) and in GDN with cAMP (right) as calculated by PDBePISA[6]. The SLC9C1 structures have been shown as a surface representation and the two protomers colored in either light orange or cyan. The oligomerization interface between the protomers is colored in ruby. **c.** left: FSEC traces of detergent solubilized crude HEK293 membranes containing SLC9C1-GFP fusion in either GDN (black trace) or DDM/CHS (purple trace), showing the shift in oligomeric stability between the different detergents. middle: Representative FSEC traces of SLC9C1 purified in DDM and CHS after incubation for 10 min at either 4°C (dotted black line) or 45°C after the addition of either DDM (gray line) or DDM solubilized synthetic lipids DOPA (olive line), DOPC (light green), DOPE (blue-green) or DOPG (purple). right: Representative FSEC traces of SLC9C1 purified in DDM and CHS including the addition of DOPA and DOPC to non-heated protein as labelled.

Supplementary Video Legends

Supplementary Video 1| Movie highlighting the fitting of the VSD domain structure in SLC9C1, obtained in the GDN detergent, into the cryo-EM map density obtained after symmetry-expansion and masked refinement of the VSD monomer. S4 helix (aqua-blue), S3 helix (silver-blue), S2 (green) and S1 (light-orange). The S4 helix is in the up-state. Gating-charge residues R803, R806, R809, located above the aromatic constriction Y743 are labelled.

Supplementary Video 2| Movie of the 3D Variability Analysis (3DVA) carried out in cryoSPARC[7] on the final cryo-EM map reconstruction of SLC9C1 in the detergent GDN processed in C2 symmetry.

Supplementary Video 3| Movie of the 3D Variability Analysis (3DVA) carried out in cryoSPARC[7] on the final cryo-EM map reconstruction of SLC9C1 in the detergent GDN and in the presence of 0.1 mM cAMP processed in C1 symmetry.

1 Boyce, K., Sievers, F. & Higgins, D. G. Simple chained guide trees give high-quality protein multiple sequence alignments. *Proceedings of the National Academy of Sciences of the United States of America* **111**, 10556-10561 (2014).
2 Brett, C. L., Donowitz, M. & Rao, R. Evolutionary origins of eukaryotic sodium/proton exchangers. *Am J Physiol Cell Physiol* **288**, C223-239 (2005).
3 Masrati, G. *et al.* Broad phylogenetic analysis of cation/proton antiporters reveals transport determinants. *Nat Commun* **9**, 4205 (2018).
4 Bazzone, A. *et al.* Investigation of sugar binding kinetics of the *E. coli* sugar/H(+) symporter XylE using solid-supported membrane-based electrophysiology. *J Biol Chem* **298**, 101505 (2022).
5 Winklemann, I. *et al.* Structure and elevator mechanism of the mammalian sodium/proton exchanger NHE9. *The EMBO journal* **39**, e105908 (2020).
6 Krissinel, E. & Henrick, K. Inference of macromolecular assemblies from crystalline state. *J Mol Biol* **372**, 774-797 (2007).
7 Punjani, A., Rubinstein, J. L., Fleet, D. J. & Brubaker, M. A. cryoSPARC: algorithms for rapid unsupervised cryo-EM structure determination. *Nat Methods* **14**, 290-296 (2017).
